# Propofol and sevoflurane induce distinct burst suppression patterns in rats

**DOI:** 10.3389/fnsys.2014.00237

**Published:** 2014-12-18

**Authors:** Jonathan D. Kenny, M. Brandon Westover, ShiNung Ching, Emery N. Brown, Ken Solt

**Affiliations:** ^1^Department of Anesthesia, Critical Care and Pain Medicine, Massachusetts General HospitalBoston, MA, USA; ^2^Department of Neurology, Harvard Medical SchoolBoston, MA, USA; ^3^Department of Neurology, Massachusetts General HospitalBoston, MA, USA; ^4^Department of Electrical and Systems Engineering, Washington University in St. LouisSt. Louis, Missouri, USA; ^5^Department of Anaesthesia, Harvard Medical SchoolBoston, MA, USA; ^6^Institute for Medical Engineering and Science, Massachusetts Institute of TechnologyCambridge, MA, USA; ^7^Department of Brain and Cognitive Sciences, Massachusetts Institute of TechnologyCambridge, MA, USA

**Keywords:** burst suppression, propofol, sevoflurane, anesthesia, rodent

## Abstract

Burst suppression is an EEG pattern characterized by alternating periods of high-amplitude activity (bursts) and relatively low amplitude activity (suppressions). Burst suppression can arise from several different pathological conditions, as well as from general anesthesia. Here we review current algorithms that are used to quantify burst suppression, its various etiologies, and possible underlying mechanisms. We then review clinical applications of anesthetic-induced burst suppression. Finally, we report the results of our new study showing clear electrophysiological differences in burst suppression patterns induced by two common general anesthetics, sevoflurane and propofol. Our data suggest that the circuit mechanisms that generate burst suppression activity may differ among general anesthetics.

## Introduction

Burst suppression is an EEG pattern characterized by quasiperiodic high amplitude activity (bursts) and relativity low amplitude activity (suppressions; Amzica, 2009; Brown et al., 2010). The phenomenon was first observed while recording EEG from the motor cortex of cats under tribromoethanol and pentobarbital-induced general anesthesia (Derbyshire et al., 1936). Investigations into the effects of ether and pentobarbital anesthesia on the EEG of canines led to the creation of the term “burst suppression” (Swank and Watson, 1949). Although early work on burst suppression focused on general anesthesia, burst suppression can be induced by several different etiologies (Martin et al., 1959).

In the first part of this article, algorithms employed to quantify burst suppression, different causes of burst suppression, and theories about the mechanisms underlying burst suppression are reviewed. We also describe clinical applications of burst suppression induced by general anesthetics. In the second part of this article, we present original research findings from our laboratory that demonstrate the distinct electrophysiological characteristics of burst suppression induced by the inhaled anesthetic sevoflurane and the intravenous anesthetic propofol.

### Quantification of burst suppression

**Figure 1:**
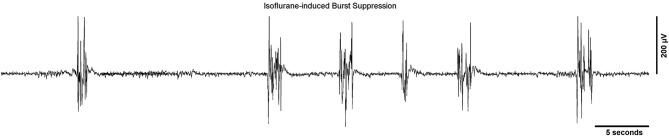
**A typical EEG recording showing a burst suppression pattern from a rodent undergoing general anesthesia from isoflurane**. The sampling rate of the signal was 512 Hz and a line filter was used to eliminate 60 Hz noise.

A widely used method for quantifying burst suppression is the burst suppression ratio (BSR; Rampil et al., 1988). Figure 1 shows several seconds of EEG burst suppression from a rodent anesthetized with isoflurane. The BSR is calculated by segmenting the EEG into bursts and suppressions using a voltage-based threshold. Suppression is commonly defined as a voltage less than 5 μV for greater than 0.5 s. This threshold is commonly set manually (Chemali et al., 2011) though automated methods such as a time-domain based voltage envelope threshold or frequency-domain based logistic regression of the EEG spectrogram have also been reported. (Prerau and Purdon, 2013; Westover et al., 2013). For the BSR algorithm, suppressions are given a value of 1 and bursts are given a value of 0 to create a binary time-series. This binary time-series is then smoothed with a windowing function to calculate the BSR over time. The value of the BSR ranges from 0 and 1, with 0 indicating no suppression and 1 indicating a suppressed EEG. Although the BSR can be derived with relative ease, the temporal resolution/smoothness of the result depends on the size of the time windows, which must be chosen manually, and the inability to obtain a measure of confidence around BSR estimates makes it difficult to perform statistical comparisons between BSR values at different points in time. Currently available EEG-based anesthetic depth monitors usually detect and quantify the BSR.

The burst suppression probability (BSP) is an alternate approach to model the level of burst suppression (Chemali et al., 2013). The BSP is based on a state-space model of the brain state of burst suppression, and represents the instantaneous probability of the brain being in a suppressed state. In contrast to the BSR, principled automated methods have been developed for setting the BSP algorithm parameters and the resulting temporal resolution/smoothness of the estimated BSP, and they also allow for statistical comparisons of the level of suppression across different points in time. Entropy measures such as approximate entropy (Bruhn et al., 2000, 2001) and machine learning methods such as artificial neural networks or support vector machines (Löfhede et al., 2007) have also been used to quantify burst suppression.

### Pathological causes of burst suppression

There are several known pathological conditions that cause EEG burst suppression. Early work with animals demonstrated that local freezing of cortical sections from cats with carbon dioxide led to profoundly decreased electrical activity, both in frozen and unfrozen areas of the brain, and that rewarming led to partial recovery of electrical activity (Nims et al., 1941). In humans, lowering of the core body temperature has been shown to linearly decrease the overall spectral power of the EEG (Levy, 1984), and burst suppression is often observed in humans with temperatures below 24.4°C (Stecker et al., 2001). Hypothermia reduces the cerebral metabolic rate, and is often used to provide neuroprotection in patients with circulatory arrest (Michenfelder and Milde, 1991; Arrica and Bissonnette, 2007).

Hypoxia is a common pathological cause of burst suppression. In animal experiments, hypoxia has been shown to induce burst suppression as well as a suppressed EEG at extremely low arterial oxygen concentrations in dogs (Spoerel, 1961). G-force induced hypoxia in rodents has also induced burst suppression (Lukatch et al., 1997). In humans, fetal hypoxia during labor and delivery can lead to hypoxic-ischemic encephalopathy in the neonate and induce burst suppression patterns in the EEG (Toet et al., 1999; van Rooij et al., 2005). While recovery from burst suppression can occur within the first 48 h after birth, the appearance of burst suppression usually portends a poor prognosis for the neonate (Grigg-Damberger et al., 1989; Hellström-Westas et al., 1995).

Patients suffering from coma may exhibit EEG burst suppression due to several different underlying etiologies (Young, 2000). Post-anoxic coma (Zaret, 1985) can induce a rare burst suppression pattern where the burst patterns are identical (Hofmeijer et al., 2014). In addition, burst suppression has been described in a survivor of post-anoxic coma during behaviorally defined sleep (i.e., eyes closed with no movement) (Kheder et al., 2014). Burst suppression may also be observed when patients are in coma due to hepatic failure (Bickford and Butt, 1955), sepsis (Young et al., 1992) and hypoglycemia (Auer et al., 1984). Coma due to porphyria, a disorder of heme synthesis (Thadani et al., 2000), can also elicit a burst suppression pattern (Dow, 1961).

Ohtahara syndrome, an early infantile epilepsy syndrome, is characterized by a burst suppression pattern that persists through behaviorally defined wake and sleep states (Ohtahara and Yamatogi, 2006). Typically Ohtahara syndrome manifests itself within 3 months of birth, and is thought to be caused by structural brain lesions. Patients with Ohtahara syndrome have been reported to have lesions of the thalamus, hippocampus, and brainstem tegmentum (Itoh et al., 2001; Ohtahara and Yamatogi, 2003). Early myoclonic encephalopathy is another infantile epilepsy syndrome that results in a persistent burst suppression pattern, usually manifesting itself during the neonatal period (Aicardi and Ohtahara, 2005). Unlike Ohtahara syndrome, early myoclonic encephalopathy is hypothesized to be due to an underlying metabolic disorder (Panayiotopoulos, 2010).

Another disorder that causes burst suppression is Aicardi syndrome, a congenital disorder in which the corpus callosum fails to develop in female infants (Fariello et al., 1977; Aicardi, 2005).In patients with a damaged corpus callosum that undergo general anesthesia, burst suppression patterns have been reported to be asymmetric and asynchronous across cerebral hemispheres (Lambrakis et al., 1999; Lazar et al., 1999).

Finally, various medications and intoxicants that are not used for general anesthesia may induce burst suppression at high doses, including ethanol (Whishaw, 1976), the muscle relaxant baclofen (Weissenborn et al., 1991; Ostermann et al., 2000), and the anticonvulsant carbamazepine (De Rubeis and Young, 2001). A recent report described burst suppression in a patient suffering from an overdose of bupropion (Mundi et al., 2012), which is used to treat depression and nicotine addiction.

### Burst suppression induced by general anesthetics

General anesthetics are administered by inhalation or intravenous injection. The main molecular targets for general anesthetics are thought to be gamma-aminobutyric acid type A (GABA_A_) receptors and N-methyl D-aspartate (NMDA) receptors (Solt et al., 2006; Brown et al., 2011), although many other targets have been identified that likely play a role in general anesthesia as well. The halogenated ethers enflurane (Lebowitz et al., 1972), isoflurane (Hartikainen et al., 1995b), sevoflurane (Scheller et al., 1990) and desflurane (Rampil et al., 1991) all induce burst suppression at sufficiently high doses. However, the haloalkane general anesthetics chloroform (Pearcy et al., 1957) and halothane (Murrell et al., 2008) have not been reported to induce burst suppression, even at high concentrations that produced suppression.

Barbiturates are intravenous anesthetics that primarily act by potentiating the function of GABA_A_ receptors. Pentobarbital (Van Ness, 1990), methohexital (Wennberg et al., 1997), and sodium thiopental (Kassell et al., 1980) are all barbiturates that have been shown to induce burst suppression. Propofol (Huotari et al., 2004) and etomidate (Modica and Tempelhoff, 1992) are not barbiturates, but they also act primarily by enhancing GABA_A_ receptor function, and also induce burst suppression. 13–15 Hz spindle activity, similar to that seen during NREM sleep, has been seen during both the burst and suppression phase of propofol-induced burst suppression (Särkelä et al., 2002; Huotari et al., 2004; Ferenets et al., 2006). Sharp waves resembling the vertex waves seen during NREM sleep have also been observed during the bursts and suppressions phases from propofol-induced burst suppression. These spindles and sharp waves are theorized to have been produced by the sensorimotor cortex (Sonkajärvi et al., 2008).

Gaseous anesthetics such as xenon or nitrous oxide that are NMDA receptor antagonists have not been shown to induce burst suppression, even at high doses in a hyperbaric chamber (Morris et al., 1955; Pittinger et al., 1955). Similarly, the intravenously administered NMDA receptor antagonist ketamine has not been shown to elicit burst suppression (Barash et al., 2012). However, the gaseous anesthetic cyclopropane, which is also an NMDA receptor antagonist (Solt et al., 2006), has been shown to induce burst suppression (Possati et al., 1953).

In summary, most general anesthetics that act primarily by enhancing GABA_A_ receptors induce burst suppression, whereas NMDA antagonists typically do not. However, there are exceptions to both rules, suggesting that molecular mechanisms alone cannot account for general anesthetic-induced burst suppression.

### Mechanisms of burst suppression induced by general anesthesia

Intracellular recordings of cortical and subcortical neurons laid the early groundwork for investigations into the mechanisms of burst suppression. While the majority of cortical cells exhibit a pattern of alternating depolarized and hyperpolarized states that account for the burst suppression pattern observed in the electrocorticogram, thalamic cells are either silent or fire at 1–4 Hz under general anesthesia (Steriade et al., 1994).

During moderate to deep levels of isoflurane anesthesia that induce burst suppression, external mechanical, visual, and auditory stimuli have been shown to trigger bursts (Yli-Hankala et al., 1993a; Hartikainen et al., 1995b; Hudetz and Imas, 2007; Amzica, 2009). Therefore, burst suppression has been considered a state of cortical hypersensitivity (Kroeger and Amzica, 2007), although external stimuli fail to induce bursting at isoflurane levels less than 2%, or greater than 3.5% (when the EEG is completely suppressed; Kroeger and Amzica, 2007). These findings suggest that the brain is still receptive to external stimuli during anesthetic-induced burst suppression. The recording of heart rate during externally triggered bursts did not show any overt changes, suggesting the effect is not derived from the autonomic nervous system (Kroeger and Amzica, 2007).

The state of cortical hypersensitivity during burst suppression is thought to be due to changing calcium levels and the lowering of cortical inhibition by isoflurane (Kroeger and Amzica, 2007; Ferron et al., 2009). Increasing the dose of isoflurane steadily lowered the amount of extracellular calcium until a state of burst suppression was reached. During burst suppression the levels of extracellular calcium decreased during bursts, and began to increase throughout the suppression period. Triggered bursts were more easily induced by external stimuli when sufficient time had elapsed after the previous stimulus, suggesting that a refractory period exists during which the extracellular calcium must reach a threshold level before a subsequent burst can be induced (Kroeger and Amzica, 2007). Administration of the NMDA antagonist MK801 significantly diminished both the amplitude and duration of bursts, but did not alter the probability of inducing a triggered burst by an external stimulus. The gap junction blocker carbenoxolone completely eliminated any triggered response, suggesting that in addition to extracellular calcium, NMDA receptors and gap junctions may also regulate the response (Kroeger and Amzica, 2007).

Phenomenological modeling of burst suppression has been performed using non-linear dynamic systems and dynamic mean field models. Modeling using chaos theory and non-linear systems for human coma patients showed that burst frequency decreased logarithmically as burst durations increased (Rae-Grant and Kim, 1994). Mesoscopic modeling using a dynamic mean field model suggested that multiple origins of burst suppression exist through several different slow modulating circuits (Liley and Walsh, 2013).

An alternative to the phenomenological models is a neuro-metabolic model, which accounts for the different etiologies that lead to burst suppression activity (Ching et al., 2012). The underlying process of burst suppression is viewed as a reduction in brain metabolism, as it is known that hypothermia, hypoxia, Ohtahara syndrome, and general anesthetics that act as GABA_A_ agonists all decrease the cerebral metabolic rate of oxygen (CMRO). The reduction of the CMRO further lowers the production rate of adenosine triphosphate, and increases cell membrane conductance. In response to lowered ATP production and increased conductance, an ATP-gated potassium channel expressed in cortical and subcortical neurons hyperpolarizes to prevent cell firing and preserve a lower energy state. This inhibition of bursting activity directly leads to the suppression period observed during burst suppression. As the suppression persists, ATP levels begin to recover and membrane conductance is lowered until another burst can occur. If the cerebral metabolic rate continues to decrease, the suppression periods will be prolonged until all bursting has ceased. This can be seen with increasing doses of general anesthetics—as the anesthetic continues to depress the cerebral metabolic rate, the EEG eventually becomes suppressed. Lowered ATP production could also lead to an impairment of calcium pumps and lead to a decrease in extracellular calcium.

This neuro-metabolic model predicts that the spectral content within bursts for a human patient undergoing propofol anesthesia will be limited to a frequency of around 10 Hz (alpha rhythm), and that this alpha rhythm can drift from having a peak power at 10 Hz at the beginning of a burst to having a peak power at 8 Hz at the end of a burst. In addition, it is thought that the spectral content of bursts reflects the neurophysiological state that was present immediately preceding burst suppression (Ching et al., 2012).

**Figure 2:**
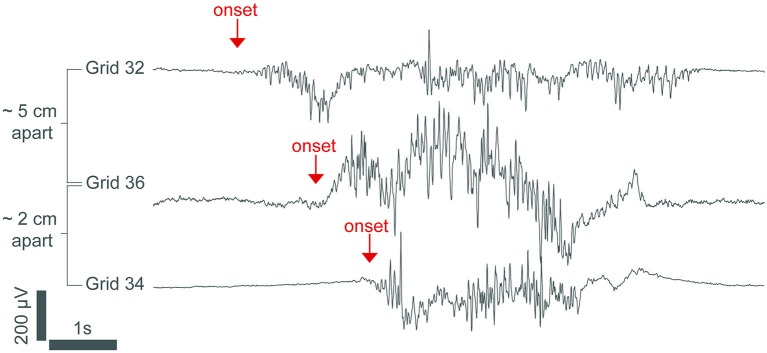
**An intracranial recording from the cortex of an epileptic patient under propofol general anesthesia**. The burst in channel 32 starts hundreds of milliseconds before the bursts in channels 36 and 34, demonstrating that burst onset is heterogeneous across the cortex. The signal was low-passed filtered at 100 Hz and resampled to 250 Hz. From (Lewis et al., 2013).

Data from human patients undergoing propofol anesthesia support this neuro-metabolic model (Lewis et al., 2013). High-density cortical recordings also revealed that burst suppression activity is not a cortex-wide phenomenon as once thought. While some regions of the cortex may be in burst suppression, other regions may not be. The occurrence of bursts can also be limited locally to discrete cortical regions. Figure 2 shows how bursts can also be spatially asynchronous across the cortex, with adjacent cortical areas having similar burst timings compared to anatomically distant areas (Lewis et al., 2013). This phenomenon was also noted in earlier human experiments (Henry and Scoville, 1952).

### Clinical applications of anesthetic-induced burst suppression

**Figure 3:**
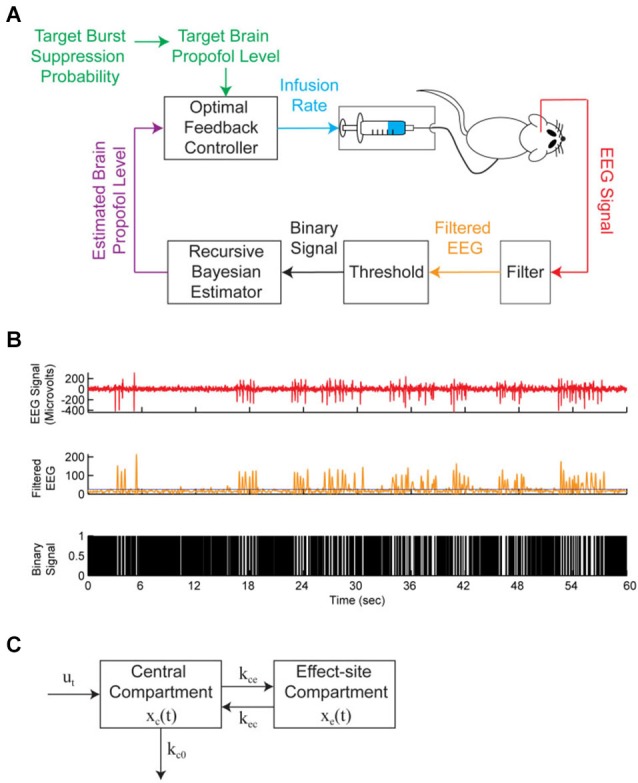
**A brain machine interface (BMI) system to control propofol-induced burst suppression. (A)** The BMI records the EEG, segments the signal into a binary time-series by filtering and thresholding, estimates the BSP or equivalently the effect-site concentration level based on the binary-time series, and then uses this estimate as feedback to control the propofol infusion rate. **(B)** A sample EEG trace showing burst suppression. The top panel shows the EEG signal, the middle panel shows the corresponding filtered EEG magnitude signal (orange) and threshold (blue) used to detect the burst suppression events, and the bottom panel shows the corresponding binary time-series with black indicating suppression events and white indicating burst events. **(C)** The two-compartmental model used by the BMI to characterize the effect of propofol on the EEG. The EEG was sampled at 500 Hz and the binary sequence was created by low-pass filtering the EEG at 5 Hz and thresholding. From (Shanechi et al., 2013b).

Status epilepticus is a state of persistent seizure activity that can last for several hours or even days (Lowenstein et al., 1999), with a mortality rate of up to 35% (Prasad et al., 2001). When status epilepticus is refractory to other therapies, seizure activity is often terminated by inducing burst suppression with intravenous general anesthetics such as propofol (Stecker et al., 1998; Prasad et al., 2001) or pentobarbital (Van Ness, 1990; Claassen et al., 2002). When treating status epilepticus, burst suppression is typically maintained by manually titrating an intravenous infusion of general anesthetic to a target BSR value. Automated closed-loop anesthesia delivery (CLAD) systems have been proposed to deliver propofol (Vijn and Sneyd, 1998) and etomidate (Cotten et al., 2011) using the BSR as the control signal. Recently, CLAD systems using the BSP as the control signal have been developed to deliver intravenous propofol in rats (Ching et al., 2013; Shanechi et al., 2013a,b), and these have been shown to achieve precise control of the level of burst suppression, obviating the need for manual titration of drug delivery. Figure 3A shows the closed-loop design of one of these CLAD systems. Figure 3B shows the process for online segmentation of the EEG for calculating the BSP, and Figure 3C shows the compartment model used to control the propofol infusion rate. Anesthetic-induced burst suppression is also used to treat patients suffering from traumatic brain injury with elevated intracranial pressures (Doyle and Matta, 1999), as well as patients suffering from severe depression (Engelhardt et al., 1993).

### A study to compare the burst suppression characteristics of two general anesthetics

Burst suppression is typically regarded as a neurophysiological phenomenon that may be caused by a range of etiologies. However, earlier experiments showed that volatile and intravenous anesthetics may have distinct electrophysiological characteristics during burst suppression. A study in rats comparing the EEG characteristics of isoflurane and propofol found significant differences between burst duration and peak-to-peak voltage at an equivalent BSR of 0.8 (Akrawi et al., 1996). However, the duration of the suppression and burst epochs that were compared were only 2–6 s. A study in rabbits comparing 1 min each of EEG burst suppression during propofol and isoflurane anesthesia reported higher amplitude bursts during isoflurane anesthesia (Hartikainen et al., 1995a). Another comparison between the burst suppression patterns of isoflurane and enflurane found that suppressions were shorter in duration for enflurane (Lipping et al., 1995). Burst suppression caused by hypoxic-ischemic encephalopathy has also been reported to have a higher variability in individual suppression durations compared to pentobarbital-induced burst suppression (Beydoun et al., 1991).

Although these reports suggest that different general anesthetics and pathological states may induce distinct burst suppression patterns, a systematic study comparing a large number of bursts and suppressions induced by two different anesthetics across all levels of burst suppression has not been performed previously. In this study, we induced different levels of burst suppression in rats with the inhaled anesthetic sevoflurane and the intravenous anesthetic propofol, and quantified the level of burst suppression using BSP. A large number of bursts and suppressions (*n* > 2000) were compared to analyze the electrophysiological characteristics of burst suppression induced by sevoflurane and propofol. We found that the durations, peak-to-peak amplitudes, and spectral power of the bursts and suppressions differed substantially between the two anesthetics at equivalent BSP levels, suggesting that at least some aspects of the mechanisms underlying burst suppression induced by sevoflurane and propofol may be distinct.

## Methods

### Animal care and use

All animal studies were approved by the Institutional Animal Care and Use Committee (IACUC) at Massachusetts General Hospital, Boston, Massachusetts. Four male Sprague-Dawley rats (Charles River Laboratories, Wilmington, MA) weighing between 550–670 g were used for these studies. Animals were provided at least 3 days of rest between experiments. Animals were kept on a standard day-night cycle (lights on at 7:00 AM, and off at 7:00 PM), and all experiments were performed during the day.

### Surgical placement of electroencephalography (EEG) extradural electrodes and recording

Rats were surgically implanted with extradural electrodes at least 7 days before experiments using previously described methods (Solt et al., 2011; Chemali et al., 2012; Ching et al., 2013). Electroencephalography was performed with a sampling frequency of 500 Hz using a QP511 Quad AC Amplifier System (Grass Instruments, West Warwick, RI), and a USB-6009 14-bit data acquisition board (National Instruments, Austin, TX). The electrical potential between stereotactic coordinates (relative to lambda) A0L0 and A6L-3 (left somatosensory cortex) was recorded. A line filter with cutoff frequencies of 0.3–50 Hz was used, and the signal was downsampled to 50 Hz.

### Preparation and delivery of drugs

Sevoflurane was obtained from Sigma-Aldrich (St. Louis, MO), and propofol (containing intralipid) was obtained from APP Pharmaceuticals (Schaumburg, IL). For the delivery of the intravenous anesthetic propofol, rats (*n* = 4) were anesthetized in an induction chamber with 2.0–3.0% isoflurane in oxygen. A 24-gauge intravenous catheter was placed in the lateral tail vein. Isoflurane was then discontinued, and the rat was removed from the chamber. After the rat fully recovered from isoflurane anesthesia, propofol was delivered using a Physio 22 syringe pump (Harvard Apparatus, Holliston, MA) until loss of righting occurred, at which time the EEG leads were attached and a rectal temperature probe was inserted. A heating pad was placed underneath the animal and used to maintain the core body temperature between 36.5° and 37.5°C.

For delivery of the volatile anesthetic sevoflurane, rats were initially anesthetized in an induction chamber with 5.0–6.0% sevoflurane in oxygen. After loss of righting occurred, EEG leads were attached and a rectal temperature probe was inserted. The rat was then placed inside a custom built anesthetizing chamber with ports for anesthetic gas delivery, scavenging, and gas sampling. A heating pad was placed underneath the chamber and used to maintain a core body temperature between 36.5° and 37.5°C. Sevoflurane concentrations were sampled and monitored from the distal end of the chamber using an Ohmeda 5250 anesthetic agent analyzer (GE Healthcare, Waukesha, WI).

### EEG recording of propofol-induced burst suppression

EEG data for propofol-induced burst suppression was taken from a previous study by our group that used a CLAD system to establish and maintain targeted BSP values using propofol (Ching et al., 2013). For this experiment, the BSP levels of 0.4, 0.65, and 0.9 were targeted in each rat (*n* = 4). Each BSP level was maintained with propofol for at least 15 min, with 10-minute ramps to transition to new BSP levels. The system used custom software initialized with MATLAB (Mathworks, Natick, MA) and issued commands to a Physio 22 syringe pump (Harvard Apparatus, Holliston, MA) using an RS-232 serial connector. The typical duration of each experiment was between 80 and 90 min. For this study, we selected 1000 s of artifact-free EEG data from each rat to provide direct comparisons with sevoflurane-induced burst suppression at equivalent BSP values in the same animals.

### EEG recording of sevoflurane-induced burst suppression

For sevoflurane-induced burst suppression recordings, the same rats (*n* = 4) from the propofol CLAD study were used. Once the animal was in the anesthetizing chamber, the dose of sevoflurane was initially set at 3.6% in oxygen with a fresh gas flow rate of two liters per minute. The sevoflurane concentration was increased by 0.2% every 30 min until a final concentration of 4.2% was reached. This maximal dose was maintained for an additional 30 min. The typical experiment duration was 120 min, and 1000 s of artifact-free EEG data was selected from each rat for analysis.

### Identification of EEG bursts and suppressions

Bursts and suppressions from the recorded EEG were segmented using a threshold based on visual inspection. Each EEG recording (*n* = 8, 1000 s each) was detrended and smoothed by convolution with a Gaussian function, and the energy was calculated using the nonlinear energy operator (Kaiser, 1990). The nonlinear energy operator provides a method for clearly separating the larger energy bursts from the lower energy suppressions, and a visually-based threshold was set in the energy domain to segment the data. The EEG values that were above the threshold were classified as bursts, whereas the values that fell below the threshold were classified as suppressions. All segmentations were confirmed by one of the authors who is an experienced clinical electroencephalographer (MBW).

### Calculation of BSP

EEG segments were converted to a binary time series. Segments that were classified as bursts were given a value of one, and those that were classified as suppressions were given a value of zero. The BSP algorithm used this binary time-vector to find the instantaneous probability of burst suppression, and corresponding confidence intervals (Chemali et al., 2011; Ching et al., 2013). Like the BSR, a burst suppression probability value of 1 indicates a state of complete EEG suppression, while a value of 0 indicates no suppression. Individual bursts and suppressions from the propofol and sevoflurane EEG datasets were sorted by their BSP into bins of 0.3–0.4, 0.4–0.5, 0.5–0.6, 0.6–0.7, and 0.7–0.8 BSP. Bursts or suppressions that were shorter than 0.15 s were discarded, as they are too short to constitute a clear burst or suppression. Table 1 gives the number of individual propofol or sevoflurane-induced bursts and suppressions within each bin.

**Table 1:** **The number of sorted individual bursts or suppressions in each BSP bin per general anesthetic**.

Propofol			Sevoflurane	
BSP	Bursts	Suppressions	Bursts	Suppressions
0.3–0.4	124	106	109	105
0.4–0.5	225	199	107	99
0.5–0.6	671	583	77	76
0.6–0.7	646	595	129	126
0.7–0.8	192	171	178	171
Total	1858	1654	600	577

*The total number of bursts was 2,458 and the total number of suppressions was 2,231*.

### Calculation of burst and suppression duration, peak-to-peak amplitude, and power

Several features of each sorted individual burst (*n* = 2,458) and suppression (*n* = 2,231) were calculated to characterize them. Using custom scripts written in MATLAB R2013b, the duration, peak-to-peak amplitude, and power of each individual burst or suppression was calculated. Duration (sec) was the absolute length of the individual burst or suppression. Peak-to-peak voltage (μV) was the absolute difference between the maximum and minimum amplitude value within each individual burst or suppression. Power (dB μV^2^/s) was the squared amplitude of the individual burst or suppression divided by its own duration.

### Statistical analysis of burst and suppression durations, peak-to-peak amplitude, and power

The median and accompanying 95% upper and lower confidence intervals for the distribution of burst and suppression durations, peak-to-peak amplitudes, and power for propofol and sevoflurane were constructed using the percentile bootstrap procedure (Efron and Tibshirani, 1993). Unlike hypothesis testing using *p*-values alone, the usage of confidence intervals gives a measure of uncertainty around the median of each feature, and testing at a 95% level is equivalent to hypothesis testing with a significance alpha of 0.05.

To make significance comparisons between the sevoflurane and propofol burst suppression features, the 95% confidence interval around the difference between sevoflurane and propofol median values was used. If the 95% confidence intervals around the differences are both positive then sevoflurane is considered to be significantly higher than propofol. If both confidence intervals are negative then propofol is considered to be significantly higher than sevoflurane. If one confidence interval is negative, and the other is positive then no statistical significance can be determined.

#### Spectral analysis of burst suppression

Spectrograms of propofol and sevoflurane-induced burst suppression were computed from EEG data using multitaper methods from the Chronux toolbox in MATLAB R2013b (Thomson, 1982; Mitra and Bokil, 2008; Babadi and Brown, 2014). Spectrograms were constructed using three tapers and a two-second window stepped through 50 ms. The half-bandwidth of the spectrogram was 1 Hz.

## Results

### Sevoflurane and propofol induce distinct burst suppression patterns

**Figure 4:**
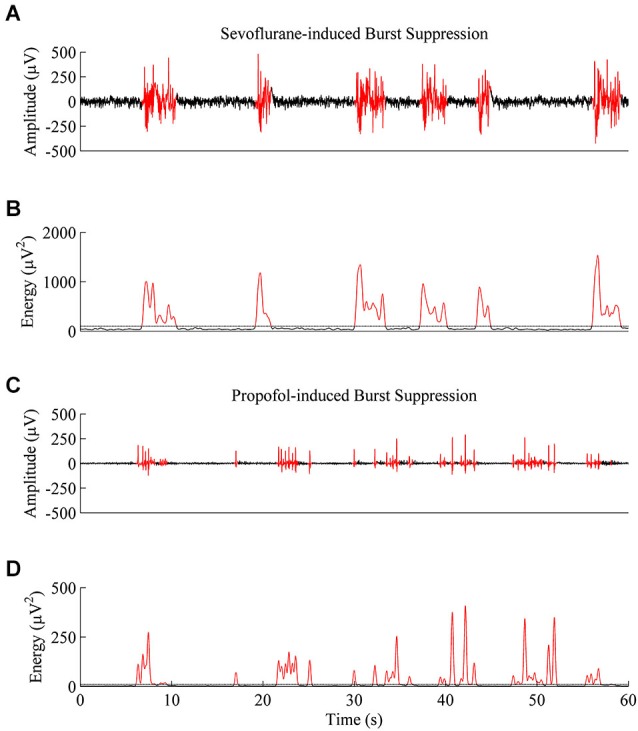
**Representative EEG traces and energy values from the same rat at a BSP of 0.7. (A)** A 60-second EEG recording taken during sevoflurane-induced burst suppression. Black indicates an area threshold as suppression, and red indicates an area threshold as a burst. **(B)** Energy of the EEG trace from **(A)** that was used to segment bursts and suppressions. **(C)** 60-second EEG recording taken from the same animal during propofol-induced burst suppression. **(D)** Energy of the EEG trace from **(C)** shows that propofol-induced bursts and suppressions are shorter and lower in power then sevoflurane-induced bursts and suppressions, despite an equivalent BSP.

Figure 4A shows 1 min of EEG data from a rat during sevoflurane-induced burst suppression at a BSP of approximately 0.7, and Figure 4B shows the non-linear energy calculated from the EEG trace in Figure 4A. Figure 4C shows 1 min of EEG data from the same rodent during propofol-induced burst suppression at a BSP of approximately 0.7, and Figure 4D shows the non-linear energy calculated from the EEG trace in Figure 4C. The visually-based threshold that was set in the energy domain to segment data into bursts and suppressions is shown as a dotted line in Figures 4B,D.

**Figure 5:**
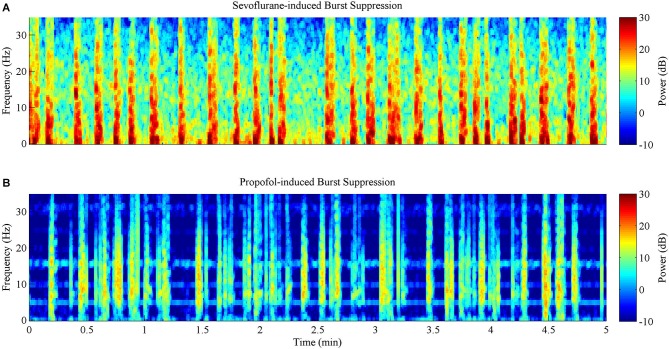
**Spectrograms computed from the same rat during 5 min of burst suppression at a BSP of 0.7**. Warm colors indicate frequency components with high power, while cool colors indicate frequency components with low power. **(A)** Sevoflurane-induced burst suppression has high power between 1–10 Hz during bursts. **(B)** Propofol-induced burst suppression has lower power during bursts and suppressions across all frequencies when compared to sevoflurane.

Figures 5A **(sevoflurane) and 5B (propofol)** show the time-frequency spectrograms for five continuous minutes of burst suppression at a BSP of 0.7 in the same rat. Warm colors (e.g., red) show areas of high power, and cool colors (e.g., blue) show areas of low power. In comparison to the burst suppression pattern induced by sevoflurane, the pattern induced by propofol was characterized by lower power across all frequency bands during both bursts and suppressions, despite equivalent BSP.

### Duration is significantly longer for sevoflurane-induced bursts and suppressions than for propofol-induced bursts and suppressions across all BSP levels

**Figure 6:**
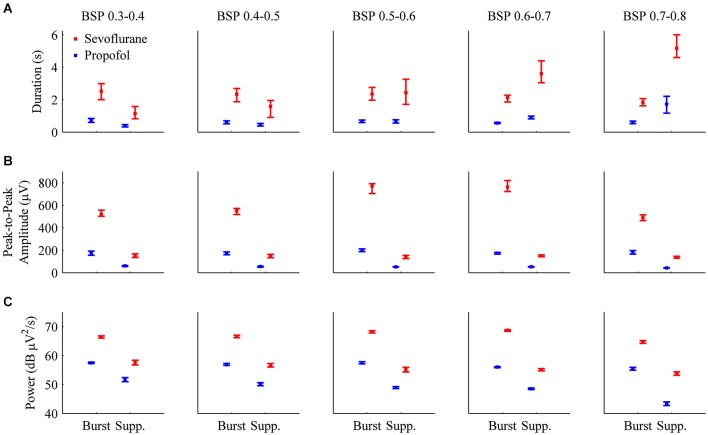
**Bar graphs with 95% confidence intervals for the median duration, peak-to-peak amplitude, and power for individual bursts and suppressions induced by propofol (blue) or sevoflurane (red) in all animals**. In order to perform direct comparisons between the two drugs at similar depths of general anesthesia, the data was grouped by BSP level. **(A)** The median durations of bursts and suppressions were significantly longer during sevoflurane anesthesia than during propofol anesthesia. **(B)** Median peak-to-peak amplitudes (μV) were significantly greater during sevoflurane general anesthesia for both bursts and suppressions. **(C)** Median power (dB μV^2^/s) for individual bursts and suppressions was significantly higher during sevoflurane general anesthesia.

Figure 6A shows the median durations for propofol and sevoflurane-induced bursts and suppressions separated by BSP. For all BSP values (0.3–0.8) the median duration of sevoflurane-induced bursts and suppressions was greater than the median duration of propofol-induced bursts and suppressions. Table 2 shows the median burst and suppression durations at all BSP levels (0.3–0.8) with corresponding 95% confidence intervals. The maximum median difference between propofol and sevoflurane bursts was 1.79 s at a BSP of 0.3–0.4, and the maximum median difference between propofol and sevoflurane suppressions was 3.46 s at a BSP of 0.7–0.8. The minimum median difference between propofol and sevoflurane bursts was 1.26 s at a BSP of 0.7–0.8, and the minimum median difference between propofol and sevoflurane suppressions was 0.76 s at a BSP of 0.3–0.4. All of the confidence intervals around the difference of medians were greater than 0, indicating that sevoflurane bursts and suppressions were significantly longer in duration across different BSP levels when compared to propofol.

**Table 2:** **The median differences between propofol and sevoflurane-induced bursts and suppressions for duration, peak-to-peak amplitude and power across BSP values of 0.3–0.8**.

BSP	Duration		Peak-to-peak Amplitude		Power	
	Burst	Suppressions	Burst	Suppressions	Burst	Suppressions
**0.3–0.4**	1.79 s (95% CI: 1.33–2.27 s)	0.76 s (95% CI: 0.41–1.29 s)	349.77 μV (95% CI: 322.16–386.61 μV)	90.09 μV (95% CI: 74.04–107.76 μV)	65.82 dB μV2/s (95% CI: 65.37–66.30 dB μV2/s)	56.14 dB μV2/s (95% CI: 55.00–57.24 dB μV2/s)
**0.4–0.5**	1.76 s (95% CI: 1.27–2.11 s)	1.14 s (95% CI: 0.41–1.46 ss)	378.46 μV (95% CI: 347.70–407.31 μV)	94.20 μV (95% CI: 80.06–109.29 μV)	66.09 dB μV2/s (95% CI: 65.59–66.57 dB μV2/s)	55.24 dB μV2/s (95% CI: 54.63–56.33 dB μV2/s)
**0.5–0.6**	1.68 s (95% CI: 1.29–2.8 s)	1.79 s (95% CI: 1.06–2.66 s)	571.07 μV (95% CI: 506.36–601.20 μV)	84.20 μV (95% CI: 72.80–102.86 μV)	67.88 dB μV2/s (95% CI: 67.34–68.23 dB μV2/s)	53.89 dB μV2/s (95% CI: 52.97–54.89 dB μV2/s)
**0.6–0.7**	1.55 s (95% CI: 1.28–1.76 s)	2.73 s (95% CI: 2.16–3.53 s)	587.73 μV (95% CI: 553.49–646.48 μV)	97.86 μV (95% CI: 89.80–105.98 μV)	68.46 dB μV2/s (95% CI: 68.07–68.65 dB μV2/s)	53.99 dB μV2/s (95% CI: 53.50–54.45 dB μV2/s)
**0.7–0.8**	1.26 s (95% CI: 1.02–1.47 s)	3.46 s (95% CI: 2.69–4.36 s)	305.02 μV (95% CI: 274.58–334.39 μV)	93.75 μV (95% CI: 85.85–102.54 μV)	64.07 dB μV2/s (95% CI: 63.63–64.72 dB μV2/s)	53.65 dB μV2/s (95% CI: 52.71–54.02 dB μV2/s)

*95% confidence intervals around the differences indicate if there is a significant increase, decrease, or no change between the two anesthetics. Sevoflurane-induced bursts and suppressions are significantly greater in magnitude than propofol-induced bursts and suppressions across all BSP values for duration, peak-to-peak amplitude, and power*.

### Peak-to-peak amplitude is significantly higher for sevoflurane-induced bursts and suppressions than propofol-induced bursts and suppressions across all BSP levels

Figure 6B shows the median peak-to-peak amplitudes for propofol and sevoflurane-induced bursts and suppressions separated by BSP. For all BSP values (0.3–0.8) the median peak-to-peak amplitudes of sevoflurane-induced bursts and suppressions was greater than the median peak-to-peak amplitudes of propofol-induced bursts and suppressions. The median burst and suppression peak-to-peak amplitudes at all BSP values (0.3–0.8) with corresponding 95% confidence intervals are given in Table 2. The maximum median difference between propofol and sevoflurane burst peak-to-peak amplitudes was 587.73 μV at a BSP of 0.6–0.7, and the maximum median difference between propofol and sevoflurane suppression peak-to-peak amplitudes was 97.86 μV at a BSP of 0.6–0.7. The minimum median difference between propofol and sevoflurane burst peak-to-peak amplitudes was 305.02 μV at a BSP of 0.7–0.8, and the minimum median difference between propofol and sevoflurane suppression peak-to-peak amplitudes was 84.20 μV at a BSP of 0.5–0.6. All of the confidence intervals around the difference of medians were greater than 0, indicating that sevoflurane bursts and suppressions were significantly greater in amplitude across different BSP levels when compared with propofol.

### Power is significantly higher for sevoflurane-induced bursts and suppressions than propofol-induced bursts and suppressions across all BSP levels

Figure 6C shows the median power for propofol and sevoflurane-induced bursts and suppressions separated by BSP. For all BSP values (0.3–0.8) the median power of sevoflurane-induced bursts and suppressions was greater than the median power of propofol-induced bursts and suppressions. Table 2 shows the median burst and suppression powers at all BSP values (0.3–0.8) with corresponding 95% confidence intervals. The maximum median difference between propofol and sevoflurane burst powers was 68.46 dB μV^2^/s at a BSP of 0.6–0.7, and the maximum median difference between propofol and sevoflurane suppression powers was 56.14 dB μV^2^/s at a BSP of 0.3–0.4. The minimum median difference between propofol and sevoflurane burst powers was 64.07 dB μV^2^/s at a BSP of 0.7–0.8, and the minimum median difference between propofol and sevoflurane suppression powers was 53.65 dB μV^2^/s at a BSP of 0.7–0.8. All of the confidence intervals around the difference of medians were greater than 0, indicating that sevoflurane bursts and suppressions were significantly larger in power across different BSP levels, when compared with propofol.

## Discussion

Previous studies on burst suppression induced by general anesthetics have found differences between burst and suppression durations and peak-to-peak amplitudes between propofol, etomidate, thiopental, and isoflurane in rodents, and between propofol and isoflurane in rabbits. The inhaled anesthetic isoflurane was found to produce greater amplitudes and durations than the other intravenous agents. However, these studies only compared a small number of individual bursts and suppressions, and did not systematically examine them at different depths of general anesthesia.

In this study, we gathered large amounts of EEG data during sevoflurane and propofol anesthesia from the same animals, and used the BSP to quantify anesthetic depth. We found that the durations of suppressions and bursts induced by propofol were significantly shorter than those induced by sevoflurane at all measured levels of BSP. Additionally, the peak-to-peak amplitudes of propofol-induced suppressions and bursts were significantly lower than those induced by sevoflurane at all measured levels of BSP. Sevoflurane suppressions were not completely suppressed, as the peak-to-peak amplitudes of sevoflurane suppressions were similar in size to the peak-to-peak amplitudes of propofol bursts. However, it should be noted that for these experiments, we analyzed EEG data at BSP levels ranging from 0.3–0.8. We did not compare burst suppression patterns at BSP levels below 0.3, due to the difficulty of visually segmenting propofol-induced bursts and suppressions at low BSP levels.

Experiments using the NMDA receptor antagonist MK801 during isoflurane-induced burst suppression showed that peak-to-peak amplitudes and durations of bursts were diminished compared to bursts induced by isoflurane alone, although the rate of bursting remained the same (Kroeger and Amzica, 2007). Nitrous oxide is an NMDA antagonist (Jevtović-Todorović et al., 1998) that decreases both suppression durations and burst amplitudes when used as an adjunct to isoflurane general anesthesia (Yli-Hankala et al., 1993b; Porkkala et al., 1997). These studies suggest that NMDA receptors play an important role in limiting the maximum amplitude of bursts and suppressions. However, in the present study we found that sevoflurane induced greater durations and amplitudes for both bursts and suppressions when compared to propofol, even though sevoflurane inhibits NMDA receptors, and propofol is thought to act primarily via GABA_A_ receptors (Solt and Forman, 2007). Our results demonstrate that NMDA receptor pharmacology alone does not account for the different burst suppression patterns observed with sevoflurane and propofol.

Extracellular calcium or ATP reuptake may also modulate the durations of bursts and suppressions. An increase in the rate of ATP reuptake under propofol (when compared to sevoflurane) could increase the rate of switching between bursts and suppressions. Cerebral blood flow could also be an important factor that determines the duration of bursts and suppressions (Kroeger and Amzica, 2007; Ching et al., 2012). It has also been suggested that during the state of burst suppression the cortex is more sensitive to external stimuli, since such stimuli have been shown to trigger bursts under isoflurane anesthesia (Hudetz and Imas, 2007). The cortex may be more sensitive to external stimuli during propofol-induced burst suppression compared to sevoflurane-induced burst suppression, allowing bursts to occur with greater frequency (Ferron et al., 2009). Despite equivalent global reduction in the CMRO, regional variations in CMRO reduction could account for the differences in burst suppression patterns observed between two different general anesthetics (Akrawi et al., 1996; Ching et al., 2012). *In vitro* studies of thiopental, propofol, and isoflurane show that these anesthetics potentiate GABA_A_ receptors. The activation of these receptors leads to a burst suppression pattern, and further increasing the anesthetic concentration depresses glutamatergic transmission. This decrease in glutamatergic transmission will eventually lead to complete suppression of the EEG. (Lukatch and Maciver, 1996; Lukatch et al., 2005).

Traditionally, the period of suppression is thought to be one of electrical silence. In the present study at 0.5 BSP, the median peak-to-peak amplitude of sevoflurane suppressions was 136 μV (95% CI: 125–155 μV), whereas the median peak-to-peak amplitude of propofol suppressions was only 52 μV (95% CI: 51–53 μV). In fact, the median peak-to-peak amplitude of propofol *bursts* (196 μV, 95% CI: 187–213 μV) was similar in magnitude to the median peak-to-peak amplitude of sevoflurane *suppressions*. This illustrates why visual thresholding was necessary for this study.

The high suppression amplitudes that we observed for sevoflurane have also been described for another halogenated ether anesthetic, isoflurane (Akrawi et al., 1996). This suggests that a greater level of neuronal activity occurs during suppressions induced by sevoflurane and isoflurane when compared to intravenous anesthetics such as propofol and barbiturates. It is known that during urethane and xylazine anesthesia, thalamic neurons fire at a steady delta rhythm (1–4 Hz) during suppression (Steriade et al., 1994). Future studies are needed to test whether thalamic firing activity is greater during EEG suppression periods induced by inhaled ether anesthetics.

Burst suppression is generally viewed as a single phenomenon that can be induced by various pathological processes as well as general anesthetics. However, the present results demonstrate that even after controlling for the depth of general anesthesia, different general anesthetics induce very different patterns of burst suppression. Automated algorithms used to segment burst suppression need to be tuned to match specific general anesthetics by taking into account the large differences in amplitudes and durations. More studies are needed to elucidate the underlying physiology that governs the burst suppression features induced by different general anesthetics.

## Disclosure of funding

This research was supported by grants TR01-GM104948 and K08-GM094394 from the National Institutes of Health, Bethesda, Maryland.

## Conflict of interest statement

The authors declare that the research was conducted in the absence of any commercial or financial relationships that could be construed as a potential conflict of interest.
